# Social-structural factors influencing periods of injection cessation among marginalized youth who inject drugs in Vancouver, Canada: an ethno-epidemiological study

**DOI:** 10.1186/s12954-017-0159-9

**Published:** 2017-06-05

**Authors:** Jade Boyd, Danya Fast, Megan Hobbins, Ryan McNeil, Will Small

**Affiliations:** 1 British Columbia Centre on Substance Use, St. Paul’s Hospital, 608-1081 Burrard Street, Vancouver, BC V6Z 1Y6 Canada; 2 Faculty of Health Sciences, Simon Fraser University, 8888 University Dr, Burnaby, V5A 1S6 BC Canada; 3 Department of Medicine, University of British Columbia, St. Paul’s Hospital, 608-1081 Burrard Street, Vancouver, BC V6Z 1Y6 Canada

**Keywords:** Injection drug use, Injection cessation, Street youth, Harm reduction, Qualitative research, Social and economic marginalization

## Abstract

**Background:**

Injection drug use is associated with HIV and hepatitis C transmission, overdose, and other preventable harms. These harms are heightened for structurally vulnerable injection drug-using populations, as their social conditions pose barriers to safer injecting. Previous research on injection cessation has largely focused on adult drug-using populations. Little qualitative work has examined the social, structural, and environmental factors that shape periods of injection cessation among youth and young adults. Such research is essential to understanding how we can best reduce harms among this vulnerable population as they move in and out of periods of injection cessation.

**Methods:**

We conducted 22 semi-structured, qualitative interviews with street-involved young people who use drugs (SY), focused on characterizing their transitions into periods of injection cessation and perceived barriers to injection cessation. Adopting an ethno-epidemiological approach, participants who had experienced at least 6 months of injection cessation were purposively recruited from an ongoing prospective cohort study of SY in Vancouver, Canada to participate in qualitative interviews. Qualitative interview findings were triangulated with the findings of a longitudinal program of ethnographic research with SY in this setting. This ethno-epidemiological approach allowed for a more robust exploration of contextual factors surrounding drug use patterns than would be possible through traditional epidemiological methods alone.

**Results:**

Findings indicate that periods of injection cessation were influenced by access to harm reduction-informed youth-focused services, transitions in route of administration (e.g., from injecting methamphetamine to the smoking of methamphetamine), and the provision of housing and social supports (e.g., from friends, family, and care providers). Conversely, participants indicated that inadequate social supports and, for some, abstinence-focused treatment methods (e.g., 12-step programs), impeded efforts to cease injecting.

**Conclusions:**

To reduce harms, it is imperative to reorient attention toward the social, structural, and spatial contexts that surround injection drug use and shape periods of injection cessation for SY. There is an urgent need for more comprehensive youth-focused services for those engaged in injection drug use, and further study of innovative means of engaging youth.

## Background

Injection drug use is associated with the transmission of blood-borne viruses such as HIV (human immunodeficiency virus) and hepatitis C (HCV), fatal and non-fatal overdose, and other preventable harms [1–4]. It is recognized, however, that many of the risks and harms accompanying injection drug use do not come from the actual act of injecting. Rather, research shows that social, structural, and environmental factors create the conditions that shape harmful injection drug use practices (e.g., syringe sharing) and risk (e.g., of overdose) [5, 6]. The risks and harms associated with injection drug use are heightened for structurally vulnerable populations [7, 8], as the social and structural inequities shaping their lives make safer injecting difficult.

The reduction of harms associated with injection drug use represents an important public health goal. Harm reduction approaches related to injection drug use in Canada include the promotion of non-injection routes of drug administration such as intranasal, oral, or inhalation drug use, as well as the transition to opioid agonist therapies (OAT) such as methadone and buprenorphine-naloxone (Suboxone). Route transitions can serve to reduce the health risks associated with injection drug use such as overdose, vein damage, bacterial infection, and blood-borne virus transmission [9–12].

Research and public health interventions attentive to the mitigation of harms associated with injection drug use have typically centered on individual behavioral change [6, 13]. However, the growing prominence of social ecological approaches such as Rhodes’ risk environment framework are evidence of an increasing appreciation of how factors exogenous to the individual shape risks and harms, as well as attempts to reduce those risks and harms [13]. The risk environment framework prioritizes examination of the social, structural, and environmental factors operating across micro- (e.g., drug use settings), meso- (e.g., institutional), and macro- (e.g., societal) levels, which shape drug-related practices and behaviors [5, 13, 14]. Indeed, previous qualitative studies examining injection cessation have highlighted the importance of social and structural factors such as peer norms and treatment programs including methadone maintenance therapy [15–18], housing, drug prohibition and incarceration [18–22], and environmental factors such as injection setting and neighborhood characteristics [23, 24].

Though early intervention has been found to be an important factor in mediating the harms associated with injection drug use, studies examining injection cessation have largely focused on adult people who inject drugs (PWID) rather than young people [19]. Increased focus on the injection drug use patterns of young people is critical, as young PWID in Canada experience numerous negative health and social outcomes. These include unstable housing [25], involvement in dangerous income generation activities such as drug dealing and survival sex work [26], increased vulnerability to police and criminal justice encounters [27], and higher mortality rates in comparison to the rest of the Canadian population [28, 29]. In the province of British Columbia (BC), drug overdose deaths for young people between the ages of 10 and 18 years old doubled from 2015 to 2016, and overdose deaths of young people from ages 19 to 29 increased from 116 to 202 people in the same time period [30]. Notably, a report that examined youth drug overdose death in BC indicated that Indigenous youth between ages 13 and 23 were significantly over-represented between 2009 and 2013 [31]. Due to dramatic increases in opioid-related drug overdose deaths in BC, the Provincial Health Officer declared a public health emergency in April 2016 [32, 33]. Vulnerable street-involved young people who use drugs (SY; defined as those temporarily or absolutely without housing or accessing street youth services) are particularly vulnerable to these harms.

Vancouver, Canada, is the site of well-established illicit drug scenes, located in the Downtown Eastside (DTES) and Downtown South neighborhoods. The DTES is a particularly stigmatized and impoverished neighborhood in the city’s downtown core, characterized by a highly visible street-based drug scene (including drug use, the selling of illegal drugs and other street-based income generation, and homelessness) [34]. This setting is also unique in terms of the high concentration of addictions services located there. Available services include harm reduction-informed programs such as needle exchange programs, methadone maintenance therapy (MMT) and Suboxone programs, and the provision of take home opioid antagonist (naloxone) kits [35]. The city is home to two federally approved supervised injection facilities (Insite and the Dr. Peter Centre), several temporary emergency overdose prevention sites located in DTES, and two harm reduction-informed detox facilities (Onsite and Directions Youth Detox). Abstinence-based programs (e.g., 12-step programs) are also accessible, including in residential settings such as recovery houses and detoxification (detox) and drug treatment facilities. In comparison to harm reduction informed programs and facilities, these programs and settings tend to have more rigid and structured policies, and prohibit any use of illicit drugs [35].

While downtown Vancouver is characterized by a high concentration of addiction services, young people in this setting nevertheless continue to experience several unique barriers to accessing these services. As has been found elsewhere across North America, these barriers include long wait times, age restrictions, experiences of discrimination based on gender, race, and sexuality, a lack of trained providers and access to OAT [36–41]. There are also significant gaps in addictions services coverage locally, including a dire shortage of dedicated residential treatment beds for young people [41]. In terms of the broader landscape in which these services are embedded, a lack of affordable and appropriate housing for youth in the City of Vancouver (one of the most expensive cities in North America to live in) has created a significant youth homelessness problem. In 2016, Vancouver’s homeless census identified 191 homeless youth under age 25 [42], with one youth shelter reporting that over 1400 young people access their services each year, a number that may be more representative [43]. Youth-dedicated community-based services, including drop-in centers, shelters, and food programs are under-resourced [41]. For Vancouver’s SY, homelessness has been found to be a predictor to injection drug use initiation [44]. Research in Vancouver has further documented high rates of syringe sharing among SY, particularly among young women, placing this population at high risk of blood borne infections and overdose [45].

This ethno-epidemiological study draws upon qualitative interviews, informed by longitudinal ethnographic work, to explore how SY in Vancouver with a history of injection drug use characterized their transitions into periods of injection cessation, as well as perceived barriers to injection cessation in this setting. In the context of semi-structured, in depth interviews, in which study participants were asked about the circumstances surrounding periods of injection cessation, SY tended to emphasize the importance of everyday conditions in their lives, rather than the specific mechanisms through which they achieved injection cessation. In this paper, we are therefore careful to locate SY’s experiences with injection cessation within the broader social, structural, and environmental contexts that powerfully shaped drug-related behaviors, decision-making, risks, and harms among these individuals.

## Methods

Participants for this ethno-epidemiological study were recruited from the At-Risk Youth Study (ARYS), a prospective cohort of street-involved and drug-using youth that has been described in detail elsewhere [46]. Initial recruitment of study participants takes place via community outreach and drop-in at a storefront research office located in Vancouver’s Downtown South. Participants are encouraged to refer their peers to the study, and health and social services professionals can also refer their clients to the study. ARYS cohort participants are between 14 and 26 years of age at the time of enrolment, and self-report the use of illicit drugs other than or in addition to cannabis during the past 30 days. Baseline and biannual follow-up visits consist of interviewer-administered questionnaire and blood tests for HIV and HCV antibodies. Pre- and post-test counseling is provided, and referrals to service agencies are available by request or at the discretion of interviewers. The baseline and follow-up questionnaires collect data pertaining to experiences with accessing addiction services, both across SY’s lifetimes and during the previous 6 months [47].

For this study, we purposively recruited 22 ARYS cohort participants who had a history of injection drug use, and had reported at a biannual visit the cessation of injection drug use for at least one 6-month period, though participants may have continued to use non-injectable drugs. The ARYS cohort database was queried in January 2013 in order to identify eligible participants. Phone calls, emails, and social media messages were used to contact eligible participants. Youth who were interested in participating in the study (i.e., who were willing to speak about experiencing periods of injection cessation) had further study details explained to them by ARYS staff and provided informed consent prior to being enrolled in the study. Semi-structured interviews took place with enrolled participants in two waves between May 2013 and September 2015. A first wave of interviews with 13 participants occurred from May 2013 to August 2013 and was conducted by the third author (MH). These interviews focused on the social, structural, and environmental factors that shaped youth’s experiences with injection cessation. A second wave of interviews with nine participants occurred from June 2015 to September 2015 and was conducted by the first author (JB). These interviews were designed to further examine key themes (e.g., access to drug treatment services, experiences with MMT programs, housing availability) that emerged during the first wave of interviews and initial analysis of interview data.

All interviews took place at our frontline research office in the Downtown South, were audio-recorded, transcribed verbatim, and checked for accuracy. All identifying information was removed from transcripts. Semi-structured interviews lasted for 45–60 min, and sought to elicit discussion of participants’ perspectives regarding their experiences with achieving periods of injection cessation. The interview guide included questions about how SY’s drug use trajectories intersected with experiences of street-based homelessness and housing transitions, and access to various services and other kinds of supports (e.g., family and friends). We asked young people to describe transitions in routes of drug administration across time (e.g., from intranasal, oral or inhaled routes of administration to injection drug use, and vice versa), and about the ways that they reduced risks and harms in relation to their drug use across time. Finally, we probed for the different kinds of barriers and facilitators young people experienced when attempting to achieve periods of injection cessation.

The methodology employed in this study reflects an ethno-epidemiological approach, in which quantitative cohort data is utilized alongside qualitative findings from in-depth interviews and long-term ethnographic fieldwork. This “value added,” mixed-method approach [48] allows for a more robust exploration of the contextual factors surrounding drug use patterns than traditional epidemiological methods alone. It results in more nuanced, participant-centered, and experiential explorations of the production of particular events and health outcomes [49–51]. In what follows, we draw upon insights gained from previous qualitative research conducted by other authors (RM, WS), which examined experiences with addiction treatment programs among local people who use drugs [52, 53], as well as an ongoing program of ethnographic research with a subsample of ARYS participants conducted by another author (DF) [54, 55]. These broader qualitative and ethnographic research findings were used during analysis to triangulate the findings of our in-depth interviews (which are the focus of this paper) and to ensure the reliability of the analysis [48].

At the time of the interview, participants ranged in age from 20 to 31, with a mean average age of 26. Eight of the interview participants identified as women and 14 as men. The majority of interviewees were white, with three young people identifying as Indigenous. Taken as a whole, the participants of this study represent a highly marginalized subpopulation of “at risk youth” and most returned to injection drug use fairly quickly after their 6-month cessation. Along with drug use, many experienced mental health issues, disability, disease, homelessness, and unstable housing. Most had low levels of education and subsisted upon income assistance, disability benefits, and informal forms of income generation such as sex work and drug dealing. Participants’ accounts often contained references to difficult childhoods and a lack of familial/social supports. A number reported frequent encounters with police.

In order to analyze the data collected, an initial coding framework developed was generated that captured emergent themes (e.g., ‘addiction treatment programs,’ ‘family support,’ ‘housing,’ and ‘MMT programs’). The analysis and coding framework was then refined using insights gained through our previous qualitative and ethnographic research, as well as through consideration of how the risk environment framework could be meaningfully applied to our data [13, 56]. All study participants received a $30 honorarium for their participation in qualitative interviews. The study received ethical approval from the University of British Columbia Research Ethics Board.

## Results

Participants’ experiences with achieving periods of injection cessation were diverse. While some described slowly reducing the frequency of injection drug use, others described quitting injecting “cold turkey.” Whether SY worked to slowly reduce injection drug use or quit all at once, for many young people periods of injection cessation were accomplished independent of engagement with addiction services, via a transition to a new route of administration and/or a new drug (e.g., from injection heroin to the smoking of heroin, or substitution with crystal methamphetamine or cannabis). For other SY, accessing various addiction services, including MMT programs, residential detoxification, and drug treatment programs, was a key pathway to injection cessation. Access to social support and stable housing was also critical for many SY as they attempted to transition away from injection drug use. Participants’ experiences with accessing addiction services, housing, and social support point to a number of important social, structural, and environmental barriers to injection cessation in our setting.

### Accessing OAT: methadone maintenance therapy

A majority of participants indicated that the primary means through which they ceased injecting drugs, specifically opioids, was by accessing OAT, in particular, MMT. Getting on MMT was also identified by SY as essential to *maintaining* injection cessation over time. However, the cost of daily dispensing fees was cited by a number of youth as a barrier to accessing MMT. For example, one participant (who began smoking heroin at age 17, and injecting it at the age of 22) explained:

I always wanted to get on methadone ‘cause I had tried rehab many times and it just didn’t work for me – like I think the longest I’ve ever lasted was like twenty-two hours or something in a place – so I always wanted to get on methadone. I had a really hard time getting on it ‘cause I wasn’t on welfare and so you have to pay. (Participant #2, White woman, 29 years old)

At the time of the interview in British Columbia, the cost of MMT was only covered for individuals on income assistance, which this young woman was initially unable to access. Ultimately, this young woman’s father ended up paying the daily dispending fee, which finally allowed her to access to MMT at the age of 25. Having access to MMT, and later income assistance, she was able to cease injecting.

### Accessing residential detox and drug treatment programs

The majority of participants expressed a similar view to that of the young women quoted above, namely, that residential drug treatment programs did not “work” for them or, youth suspected, many others:

Ninety-five percent of the people like in treatment centres that I’m seeing, are relapsing and– like this system is not working and it’s getting a lot of money. (Participant #17, White male, 30 years old)

Participants frequently described negative experiences while in abstinence-based residential detox and drug treatment programs, including, for example, overly restrictive rules and regulations (e.g., the requirement to attend daily 12-step meetings and group counseling sessions, and “one strike and you’re out” rules that meant expulsion from the program for any form of drug use). The residential detox and drug treatment programs available to young people in Vancouver vary dramatically in terms of quality and philosophies of care. While abstinence-based programs (usually informed by the 12-step model) were reported by a small number of participants to be beneficial in terms of facilitating short-term injection cessation, they were usually characterized as being insufficient to support long-term cessation of injecting. One participant noted that the 12-step program they attended worked initially but eventually did not facilitate their injection cessation:

I went to lots of [12-step] meetings. I don’t really go anymore but I went to a lot. I got a sponsor. I did the step work and it really helped in the beginning, it was a good stepping-stone. (Participant #4, White woman, 28 years old)

Consistent with the findings of our longitudinal ethnographic work, participants’ access to residential detox and drug treatment programs was limited by long waitlists, as well as experiences of aging out of youth-focused addiction services, which are capped at ages ranging from 18 to 24 years of age. One 23-year-old man described employing a combination of Suboxone (which was not readily available through, or covered by, pharmacare at the time of data collection) and his own methods (i.e., biking, yoga) to achieve and maintain injection cessation, because he was too old to access the harm reduction-informed youth detox he had benefited from previously:

Like you go there to detox but people are there to take care of you and it’s beautiful. And you’re able to go smoke, like whenever you ask can I go for a smoke and they brought you smokes and I love that place. It’s amazing for youth under 21. I wish there was a thing like that for uh [older people]. (Participant #15, White man, 23 years old)

Our long-term ethnographic research indicates that aging out of youth-specific services presents a particular problem for young people who are generally unwilling to access residential detox and drug treatment programs located in Vancouver’s DTES neighborhood, which are inclusive of all ages.

### Achieving periods of injection cessation independent of addiction services engagement

Some participants appreciated the rules and structure that abstinence-based detox and drug treatment programs offered. However, a majority of study participants indicated that they eventually avoided abstinence-based detox and drug treatment programs as a result of what they characterized as restrictive policies (i.e., expulsion for drug use, missing curfews, house chores, meeting attendance, and noise complaints). These youth generally came to view injection cessation as something that they had to accomplish on their own, through what they referred to as “their own methods of harm reduction.” For many participants, periods of injection cessation were facilitated via different kinds of transitions in drug use. Some young people who quit injecting drugs continued to use non-injection illicit drugs. For example, a small number of youth transitioned from injecting to smoking crystal methamphetamine in an explicit attempt to reduce harms. Alternatively, several participants identified cannabis as means of assuaging the feelings of discomfort that accompany quitting injection drug use:

Being on drugs for such a long period of time is like, that’s how you’re normal […] and now when I stopped using, like, I’m still having a hard time adjusting, […] it’s really hard to deal with and that’s why I started smoking weed…. (Participant #11, Indigenous woman, 27 years old)

### Accessing harm reduction-informed services

Participants also often emphasized the value of accessing harm reduction-informed services in aiding their eventual transition away from injecting. Harm reduction-informed services, including drop-in centers, shelters, and two local detox facilities, generally encourage youth to inject more safely (i.e., by providing sterile injecting paraphernalia and safer injecting education) or to take a short-term break from injecting, with the offer of additional support (e.g., residential drug treatment placements) if and when youth decide to transition away from injecting more permanently. In comparison to abstinence-based detox and drug rehabilitation programs, and in particular, those informed by the 12-step model, these service spaces were described by SY as less judgmental toward those who were gradually moving toward injection cessation as a means of reducing harms in their lives. One young woman compared her experience at the harm reduction-informed youth detox, which she described as “low key,” with an “absolutely terrible experience” she had at an abstinence-based, more medicalized detox, stating definitively, “I just don’t like medical detox”:

It’s cool that you can choose your own food. Like they’ll be like hey, you know are you getting hungry? Oh like, you want me to make you this or what do you want? They’ll take you to the park if you wanna like just be a kid and like play on the playground. Um, I dunno, they’re just cool. [….] Like there aren’t nurses running around me like here’s your meds [medication] rah rah rah, like it’s just… it’s better. (Participant #7, White woman, 20 years old)

The following participant similarly described a positive experience at Vancouver’s other harm-reduction informed detox (Onsite), which he accessed in order to meet the criteria (1 week of abstinence) for entrance into a longer-term treatment center:

I just slept most of the day, and kind of enjoyed little activities, and just eating regular food without having to stand in food lines, and did lots of fun activities, and enjoying some camaraderie there. (Participant #17, White male, 30 years old)

Both of Vancouver’s harm reduction informed detoxes are less rigid in enforcing abstinence, and instead of working with clients on a case-to-case basis, recognizing that hasty ejection from detox may precipitate increased instability, risk, and harm in individuals’ lives. In general, harm reduction-informed services often were felt to take a more holistic approach to supporting periods of injecting cessation by facilitating SY’s access to basic necessities and multiple supports including shelter, housing, work, clothing, showers, and food. These more “relaxed” and “respectful” service spaces (as participants often put it) were also often more conducive to the establishment of meaningful relationships with service staff; a valued source of social support for many SY.

### Social support

Participants indicated that support from health and social service providers, as well as from family and friends, could be integral to achieving and maintaining injection cessation. As described earlier, one participant was only able to access MMT with the financial help of her father. Social support was also central to the experience of another young man, who connected his positive experience in a longer-term recovery house with the relationship he formed with a service provider:

The guy who was the worker that got me there would take me to movies and [we] became friends and you know, it was just a friend. You know? (Participant #16, White man, 30 years old)

One young woman, who was court ordered to remain abstinent for 14 months due to a criminal charge related to drug trafficking, resisted formal pressures to reside in a recovery house despite her fear of imprisonment, explaining that having a home and the support of a “loving family” were more useful in maintaining injection cessation than the addiction services available to her, which she characterized as overly restrictive (Participant #14, Indigenous woman, 29 years old).

Participants who achieved periods of injection cessation as a result of steady family support were the exception, however. The support of family members and even friends was generally scarce among study participants; most expressed that they had few people in their lives that they could rely upon. Obtaining adequate support from professionals could also be a challenge. One young man described his inability to find a family doctor after being “dropped” by his former doctor because she “had too many patients,” and expressed how his isolation was a barrier to maintaining injection cessation:

Ah, I’m somewhat isolated, I would say – of support. Like I don’t have a regular counsellor. I don’t have a lot of friends. I guess it’s just like maybe if I was looking at it from the outside, I would say I don’t have the strongest support network. (Participant #17, White man, 30 years old)

One Indigenous young man (participant #9, 29 years old) stressed the importance of receiving support via culturally appropriate, non-racist services that evidence respect for diverse Indigenous customs, values, and beliefs. Several participants (both women and men) emphasized the need for additional gender-sensitive supports, such as children friendly services.

### Access to housing

SY understood stable housing to be key to their efforts to achieve and maintain injection cessation. One participant, for instance, noted that a combination of obtaining stable housing and work helped him to cease injecting heroin:

I mean it helps me still today, I’m so busy I have no time, I have no time to take it, I don’t have time to do it. [….] When I have time to myself I’m trying to, I found some new passion, um, you know I didn’t have that before, passions, because I didn’t have anything, like I couldn’t put anything anywhere. Now I have an apartment so I can put stuff in there. So if I want to go buy like a punching bag, I’ve got a place to put it. I got a yoga mat so I can do some yoga, you know. I’m able to go for a four or five hour bike ride. (Participant #15, White man, 23 years old)

When asked what services they thought might enable SY to achieve periods of injection cessation, many participants responded that they needed affordable, quality housing and access to other basic necessities such as food and education before they could even begin the process of transitioning away from injecting:

Affordable housing. I think that’s a really big one. And access to healthy food I think is a really big one too. ‘Cause most food banks and stuff like that just, it’s really terrible stuff. Like all processed and just gross. Um, [pause] and like just education I guess. Free education. (Participant #7, White woman, 20 years old)

Participants indicated that not only the quality but also the location of housing impacted their drug use. Specifically, they indicated that the ability to move further away from Vancouver’s inner city drug scene could be beneficial to achieving periods of injection cessation. For example, one young man explained why it was important to him to move away from Vancouver’s DTES:

‘Cause like the minute I go outside of my place all those people will be all messed up and on the street right? So like you gotta leave that stretch of area if you’re gonna, you know, try to get out of the drug scene. [….] I don’t even go to Hastings [Street] anymore [….] Like, I don’t even wanna go down there anymore. (Participant #20, White man, 21 years old)

Despite a desire to move out of the downtown core as a means of achieving and maintaining injection cessation, participants often lamented their *inability* to re-locate due to a lack of financial and social supports.

## Discussion

Our findings point to the multiple contexts and supports that shape SY’s abilities to achieve and maintain periods of injection cessation, as well as the various barriers they face to reducing harm in their own lives. Participants spoke of the importance of housing, social support, harm-reduction informed services, and various addiction services to injection cessation. However, they also highlighted the strategies that they employed independently of service engagement, which primarily involved transitions in the route of drug use and forms of drug substitution that ranged from MMT to the use of cannabis.

Contrary to the findings of a study conducted with young PWID in San Francisco [19], participants in this study reported that the use of cannabis helped in achieving and maintaining periods of drug use cessation. Dynamics reported by our study participants are consistent, however, with more recent research in Canada and the USA, which point to cannabis substitution to be a potentially effective harm reduction strategy for PWID [57–60]. Participants also described OAT as a useful method for achieving injection cessation and stabilizing their lives. This is consistent with previous research which indicates that OAT allow individuals to reduce injection risks, in addition to treating opioid dependence [61–64]. Significantly, among PWID, methadone therapy has been shown to reduce risk of hepatitis C and HIV infection [64, 65].

Largely consistent with previous research, participants also emphasized a range of barriers to the cessation of injection drug use. Notably, many study participants experienced barriers to accessing OAT programs such as MMT, primarily because of the daily dispensing fee, which is a problem that has been well documented in existing literature [63, 66, 67]. There have been some recent changes to opioid substitution therapy in B.C. In 2015, Suboxone treatment expanded and is now covered by B.C.’s public drug plan [68, 69]. This is an important policy shift as risk of overdose is much lower with buprenorphine treatment as compared to methadone [70, 71]. Participants explained that family and social supports also had a positive impact on the initiation and continuation of OAT and other drug treatment programs, as has been demonstrated in previous research [72]. In general, increased family responsibility and social supports have been associated with the cessation of illicit drug use [73, 74], while family pressure has been indicated as a self-reported reason for injection cessation [75].

While participants accessed a range of services, harm reduction-informed services rather than abstinence-based ones were emphasized as productive in the facilitation of periods of injection cessation. This finding supports previous research showing that abstinence-based programs are often unsuccessful and can even be counter-productive [11]. People are significantly more vulnerable to overdose following release from residential abstinence-based detox and drug rehabilitation programs, or if expelled from OAT [76–78]. The more general barriers to addiction treatment described by participants, such as waitlists, age limitations, and restrictive abstinence-based and 12-step informed policies are consistent with previous studies on youth’s access to addiction services [41, 79–82]. An epidemiological study of predictors of injection cessation in San Francisco, for example, stressed addiction services targeted at young people as an important area of intervention. Such interventions need to take into consideration the fact that vulnerable and marginalized young people dealing with substance use may be less likely to approach service providers due to issues of trust and discrimination as well as a dearth of culturally appropriate and sex/gender sensitive services [41].

Our findings further highlight that housing is an important facilitator of periods of injection cessation among SY, and should therefore be a fundamental component of health and drug policies related to youth substance use. For marginalized people in Vancouver, and in particular youth, lack of housing has adverse health and social outcomes and negatively impacts the potential for injection cessation. Our findings resonate with an epidemiological study in Baltimore, which found that stable housing is associated with shorter time to injection cessation, and additional epidemiological studies in Montreal and Chennai (India) which found that homelessness impedes injection cessation [18, 20, 75]. Safe and affordable social housing is not only a human right—it is a crucial means of reducing the harms associated with substance use and can promote social inclusion [83–88]. In BC, research demonstrates that for youth who use drugs, appropriate low-threshold housing with support is lacking, yet essential to their well-being [41]. The injection-related risks faced by street-involved young people in Vancouver is often further exacerbated by their homelessness [27, 89]. In addition, prolonged presence in micro-risk environments as a result of the inability to exit particular local drug scenes or housing settings also increases harms related to injection drug use [8, 56, 85]. In our setting and others, residence in neighborhoods associated with deprivation and “disorder” have been found to have a negative impact on injection cessation [90, 91].

This study has several limitations. The methodological approach is subject to limited generalizability and social desirability bias. However, an ethno-epidemiological approach, which incorporates longitudinal ethnographic observations into the analysis of qualitative interview data, allows for a more comprehensive interpretation of this data [48, 92, 93]. Further, the research findings are situated within a unique setting (an urban center with a number of addiction, social and health services, and harm reduction initiatives). Our findings may not be reflective of the experiences of the larger injection drug using youth community either locally or nationally. For instance, not all provinces in Canada include harm reduction in their health strategies and previous research has found that younger PWID have been denied access to MMT based on age [94]. In addition, the underrepresentation of Indigenous participants in the study points to the need for further attention to barriers to injection cessation among this vulnerable population.

## Conclusions

In conclusion, our findings highlight the broader social, structural, and environmental contexts that powerfully framed—or prevented—periods of injection cessation among highly marginalized SY. This study has implications for health and social policy. Namely, we argue that it is imperative to include the perspectives of young PWID in policy discussions and to reorient attention toward the socio-economic roots of poverty, drug policy, and related social and health harms [1, 95]. We further emphasize the need for a more comprehensive system of care for young PWID, with attention to facilitating better treatment access for young marginalized populations and developing youth oriented services attentive to diversity and youth perspectives. As well, increased consideration of the promotion of non-injection routes of drug administration, rather than more singular emphasis on abstinence, could play a significant role in the minimization of harms associated with injection drug use for young people. These final recommendations, however, will not be effective unless the underlying social structural factors that frame injection drug use among young PWID are also addressed.

## Abbreviations

ARYS: At-Risk Youth Study
B.C.: British Columbia
DTES: Downtown Eastside
HCV: Hepatitis C
HIV: Human immunodeficiency virus
MMT: Methadone maintenance therapy
OAT: Opioid agonist therapies
PWID: People who inject drugs
SY: Street-involved young people who use drugs
